# Clinical and functional outcome following robotic Heller-myotomy with partial fundoplication in patients with achalasia

**DOI:** 10.1007/s11701-023-01557-3

**Published:** 2023-03-25

**Authors:** Sebastian M. Rabe, Eva Burmeister, Stefan Niebisch, Ines Gockel

**Affiliations:** Department of Visceral, Transplant, Thoracic and Vascular Surgery, University Medical Center of Leipzig, Liebigstr. 20, 04103 Leipzig, Germany

**Keywords:** Achalasia, High-resolution manometry, Heller myotomy, Robotic, Functional outcome

## Abstract

Robotic-assisted myotomy with partial fundoplication for patients with achalasia has been established as a safe and effective procedure with similar short-term results and lower rates of intraoperative esophageal perforations. Our aim was to investigate a defined patient cohort undergoing robotic-assisted and laparoscopic surgery providing pre- and postoperative symptom score and high-resolution manometry to evaluate the clinical and functional outcome.All patients underwent clinical, endoscopic, radiological and manometric investigation to verify the diagnosis of achalasia. High-resolution manometry was performed preoperatively and 6 months postoperatively and categorized according to the Chicago Classification (v4.0). We used the Eckardt Score to evaluate symptomatic outcome. All patients underwent either robotic-assisted or laparoscopic myotomy with partial anterior fundoplication (180° Dor) using the DaVinci Xi surgical system (Intuitive, Sunnyvale, California, USA). From a total amount of 101 patients, we analyzed the data of 78 (47 robotic and 31 laparoscopic) procedures between 2015 and 2020. All patients showed a significant decrease of the Eckardt Score in the robotic group (median 6 vs. 2) as well as in the laparoscopic group (median 7.5 vs. 3). The postoperative LESP and 4 s-IRP was significantly reduced in all patients in the robotic group [median LESP (mmHg) 34.16 vs. 16.9; median 4 s-IRP (mmHg) 28.85 vs. 14.55], as well as in the laparoscopic group [median LESP (mmHg) 35.34 vs. 17.3; median 4 s-IRP (mmHg) 25.6 vs. 15.9]. There was no significant difference for these parameters between the groups. There was no event of intraoperative esophageal perforation in the robotic cohort, whereas there were 2 in the laparoscopic group. Our data support the safe and effective robotic approach for the surgical treatment of achalasia. Not only the clinical outcome but also the functional results measured by high-resolution manometry are similar to the laparoscopic procedure. Further investigations in larger prospective multicenter studies are needed.

## Introduction

Achalasia is a rare neurodegenerative disease with an incidence of 1–2/100,000 per year. The primary esophageal motility disorder is caused by a loss of inhibitory nerve cells of the myenteric plexus resulting in an absent or reduced relaxation of the lower esophageal sphincter (LES) and dysfunctional peristalsis of the tubular esophagus [1]. Key symptoms include dysphagia, regurgitation, chest pain and weight loss typically quantified by the Eckardt Score presenting the frequency and/or intensity of these features. It is, therefore, a valuable tool to assess the clinical outcome after surgical or endoscopic treatment [2]. Esophagogastroduodenoscopy is commonly performed at first to exclude secondary forms of achalasia, e.g. due to carcinoma or inflammation of the esophagogastric junction frequently followed by barium esophagogram showing the concise ‘bird’s beak sign’ or typical sigmoid-shaped esophagus in late stage disease. High-resolution manometry (HRM) is possibly the most important examination for confirmation, early detection, exclusion and follow-up of achalasia [3]. Moreover, it is a crucial element for stratification according to the Chicago Classification, which is based on manometric findings in these patients and therefore important to find the optimal therapeutic strategy, as it differs among the different subtypes [4]. The therapeutic options include medication (calcium channel blockers, nitroglycerine), botulinum toxin injection, pneumatic dilatation or peroral endoscopic myotomy. The surgical gold standard has been the minimally invasive Heller myotomy with partial fundoplication for many decades, reducing the resting pressure in the LES by dividing its longitudinal and circular smooth muscle fibres [5, 6]. Postoperative permanent improvement of dysphagia can be seen in 85–100% of patients [7, 8]. With the rise in robotic-assisted surgery in the past 2 decades due to enhanced 3D-visualization, antitremble filters and increased freedom of movement, similar short-term results with even a lower rate of intraoperative esophageal perforations have been shown [9–12]. However, due to a lack of short-term results providing clinical and functional outcomes, our aim was to investigate a collective undergoing robotic-assisted (RHM) and laparoscopic Heller myotomy (LHM) providing pre- and postoperative symptom score and high-resolution manometry.

## Patients and methods

We performed a retrospective review of patients who underwent either RHM or LHM between January 2015 and September 2020 (sequentially). Diagnosis of idiopathic achalasia was based on symptoms, esophagogastroduodenoscopy, barium esophagogram and esophageal HRM. Clinical symptoms were assessed using the Eckardt Score preoperatively and 6 months postoperatively. Its items dysphagia, regurgitation, chest pain and weight loss in kilogram are scored 0–3 points and summarized to a total score ranging from 0 to 12. No weight loss scored 0 point, ≤ 5 kg 1 point, 5–10 kg 2 points and > 10 kg 3 points. The frequency of the other 3 symptoms were each scored by the following system: none 0 points, occasional 1 point, daily 2 points and each meal 3 points. A total score of 3 points or less were considered postoperative success. Patient data are maintained in a database approved by our Institutional Review Board. All patients gave written informed consent before operation and follow-up data collection.

Esophageal HRM was performed preoperatively and 6 months postoperatively in all patients. We used the standardized study protocol for examination and calculation as previously published by Niebisch et al. [13]. Examinations were performed using software and catheters available from Medical Measurement Systems B.V. [Medical Measurement Systems, Enschede, Netherlands] and Unisensor AG [Unisensor AG, Attikon, Suisse]. For analysis, we used the most recent software version available (MMS database software, version 9.6b, March 04, 2020). Using a catheter system with 36 pressure channels placed at a distance of 1 cm, it was placed transnasally in patients fasted for at least 6 h. The catheter was placed one or two channels above the upper esophageal sphincter and at least three channels below the diaphragm marked by the pressure inversion point between the intraabdominal and intrathoracic cavity assuring a safe intragastric placement of the catheter. In cases of uncertainty, deep inspiration increased the pressure difference therefore assured the correct position. After accommodation for 5–10 min in a semi-supine position at 30° elevation, examination started with 30 s of baseline recording in normal respiration without coughing or swallowing followed by ten 5 ml water swallows every 30 s. As there are no normal thresholds following upper gastrointestinal (GI) surgery, we referred postoperative manometric findings to the normal thresholds.

According to the Chicago Classification version 4.0 [4], we subclassified three phenotypes of achalasia:

- Type I: achalasia with complete aperistalsis of the tubular esophagus
- Type II: achalasia with panesophageal compression
- Type III: achalasia with spastic contractions of the esophagus

Furthermore, demographics, previous treatments, intra- and postoperative complications, operative time and length of hospital stay were analyzed and compared between the two groups. Statistical analysis was performed using SPSS Version 28.0.1.1 (IBM SPSS Statistics; Chicago, IL, USA). Data are expressed as median and interquartile range (IQR) unless stated otherwise. Kolmogorov–Smirnov test was used to check for normal distribution. *F* test was applied to examine the equality of variances, Student’s *t* test or Mann–Whitney *U* test were used for determination of statistical significance. A *p* value < 0.05 was considered statistically significant.

### Robotic-assisted Heller myotomy (RHM) and Dor fundoplication (DF)

We performed operations using the da Vinci Xi surgical system by Intuitive Surgical, Inc. (Sunnyvale, CA, USA). The patient is in a supine position with his left arm attached to his body as the patient cart coming from the left site of the patient. To prevent from slipping, the patient is bedded in a vacuum mattress. To improve the exposition of the esophagogastric junction, the operation room table is flexed slightly to achieve an extension in the thoracic spine. A single-shot dose of antibiotics is administered 30 min before the skin incision. Preoperatively, orogastric tube with a 36 Fr diameter is placed serving as a splinting for the later esophagogastromyotomy.

Placing of the trocars is done laparoscopically starting with an 8 mm camera trocar midline supraumbilical by mini-laparotomy to establish the capnoperitoneum. We set a pressure from 12 to 14 mmHg depending on the patient’s constitution and co-morbidities. We introduce three additional 8 mm robotic trocars approximately 7 cm apart from each other in line with the camera port as well as a 12 mm assistant trocar in the right lower quadrant of the abdomen. A 5 mm subxiphoid incision is made for inserting the retraction system for the left hepatic lobe. After positioning the patient in reverse Trendelenburg, the patient cart is docked and the robotic arms are attached to the ports. Under constant visualization, the instruments are inserted into the abdominal cavity. From patient’s right to left, we use the following line up: Port 1—Force Bipolar, Port 2—camera, Port 3—monopolar curved scissors, Port 4—Cadiere Forceps or needle driver. Starting by dissection of the pars flaccida of the hepatogastric ligament, we identify the right crus of the diaphragm and continue our dissection until identification of the left crus of the diaphragm to separate the esophagus from the phrenoesophageal membranes. If necessary, to ensure a sufficient mobility of the gastric fundus for later forming of the anterior fundoplication, the short gastric vessels are dissected. After an adequate cranial mobilisation of the esophagus, the vagal nerve is safely identified and will be taken care of for the rest of the procedure. We initiate the esophageal myotomy at 11 o’clock to the left lateral site of the vagal nerve approximately 2–3 cm above the esophagogastric junction by tearing apart the muscle fibres (bluntly). We continue proximally up to 5–6 cm in the submucosal plane taking care of any residual muscle fibres that have to be dissected. We carry on distally with the dissection of the anterior gastric wall for 2–3 cm. Overall, we achieve a length of the esophagogastromyotomy measuring 7–8(9) cm.

Since we check for any residual bleeding, anterior fundoplication according to Dor (180°) is formed by placing the mobilized gastric fundus above the myotomy and attaching it with the esophageal wall and ipsilateral crus of the diaphragm with 2–3 silk sutures on each site. In case of a concomitant hiatal hernia, a ventral hiatoplasty is necessary.

### Laparoscopic Heller myotomy (LHM) and Dor fundoplication (DF)

For our laparoscopic approach, the patient is placed in the lithotomy position with both arms abducted and hyperextension in the thoracic spine. Establishing the capnoperitoneum by insertion of a 10 mm trocar by midline supraumbilical mini-laparotomy, we use 2 additional trocars subcostally approximately 10 cm below the xiphoid process in the midclavicular line on both sides as well as two 5 mm assistant trocars on each side. Then the laparoscopic procedure is performed in analogy as the previously described robotic-assisted approach.

## Results

Between January 2015 and September 2020, we performed a Heller myotomy and partial anterior fundoplication in 101 patients with achalasia. We were able to include 78 of these (47 patients in the RHM group and 31 patients in the LHM group) (sequentially, starting with the first robotic patient in 2017) with full data sets of HRIM and Eckardt Score pre- and postoperatively. All operations were performed by two experienced upper GI surgeons (IG and SN). Patients’ characteristics including age, sex, body mass index (BMI), achalasia subtype and preoperative interventions are shown in Table 1.

**Table 1:** Demographic characteristics

	Laparoscopic procedure (LHM) (*n* = 31)	Robotic-assisted procedure (RHM) (*n* = 47)	*p* value
Age (years), median (IQR)	43.5 (52.0, 66.5)	53.0 (41.0, 59.5)	0.111
Sex (*n*)			0.229
Female	18	22	
Male	13	25	
BMI (kg/m^2^), median (IQR)	24.0 (21.0, 28.0)	24.0 (22.0, 27.0)	0.774
Achalasia subtype (*n*)			
Type I	16	16	0.147
Type II	13	30	
Type III	2	1	
Preoperative interventions			
Botox (%)	9.7	4.3	0.308
Pneumatic dilatation (%)	61.3	31.9	0.01
Myotomy (%)	19.4	0	0.003

All values are given in median (IQR) unless stated otherwise

*p* value <0.05 was considered statistically significant

**Fig. 1:**
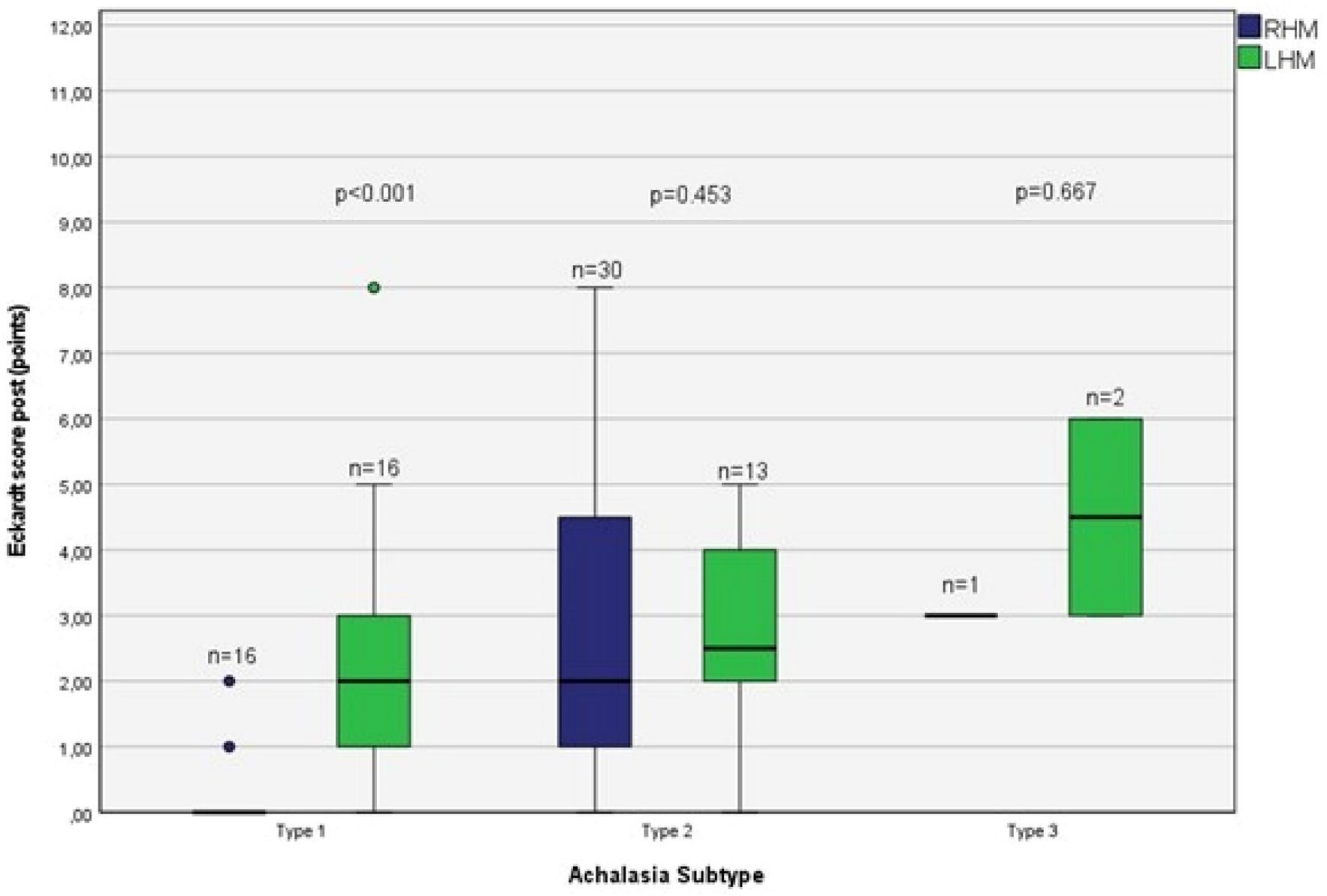
Eckardt Score postoperative (points): RHM vs. LHM

The operative time was shorter in the RHM group (median of 112.0 min) compared to LHM group (median of 117 min) without statistical significance (*p* = 0.179). The length of stay was significantly shorter in the RHM group (median of 2 days) compared to the LHM cohort (median of 3 days; *p* < 0.001). There were 2 intraoperative perforations in the LHM group (6.45%), while none occurred in the RHM group (0%). There were no cases of delayed perforations in both groups, neither occurred any major complications or postoperative deaths. Conversion to an open procedure was not necessary in either of the groups. The median follow-up period was 7 months (range 6–9 months). A significant reduction in the postoperative Eckardt Score could be shown for the RHM group (median 6 points vs. 2 points; *p* < 0.001) as well as for the LHM group (median 7.5 points vs. 3 points; *p* < 0.001) (Table 2). There was no significant difference between the two groups in the postoperative Eckardt Score (*p* = 0.299). This was also shown when divided into Achalasia subtypes for the RHM group as well as for the LHM group (Fig. 1).

**Table 2:** Comparison of pre- and postoperative parameters in groups with patients treated with RHM and LHM

	RHM (*n* = 47)	LHM (*n* = 31)	*p* value
4 s integrated relaxation pressure (mmHg) pre	28.85 (23.635, 36.3)	25.6 (13.7, 36.3)	0.170
4 s integrated relaxation pressure (mmHg) post	14.55 (7.58, 24.6)	15.9 (12.45, 23)	0.323
*p* value	< 0.001	0.019	
LES resting pressure (mmHg) pre	34.16 (27.33, 46.5)	35.34 (19.98, 47.4)	0.255
LES resting pressure (mmHg) post	16.9 (7.7, 28)	17.3 (15.35, 26)	0.494
*p* value	0.005	0.007	
Eckardt score pre (points)	6 (5.8)	7.5 (5.7)	0.459
Eckardt score post (points)	2 (1.5)	3 (1.4)	0.299
*p* value	< 0.001	< 0.001	

All values are given in median (IQR) unless stated otherwise

*p* value <0.05 was considered statistically significant

Only in patients with Achalasia type I, there was a significant reduced postoperative Eckardt Score in the RHM group compared to the LHM group. Regarding the high-resolution manometric parameters of the esophagogastric junction, a significant reduction of the LES resting pressure (LESP) as well as of the 4 s-Integrated relaxation pressure (4 s-IRP) could be detected in both groups (Table 2).

Likewise, the postoperative reduction in the LESP was also detected when divided into subtypes for the RHM group [Type I median (IQR) LESP pre (mmHg) 27.75 (15.65, 42.25) vs. post 16 (8.08, 37.28); *p* = 0.183, Type II pre 35 (28.85, 47.3) vs. post 17.15 (8.38, 32.55); *p* = 0.002], as well as for the LHM group [Type I median (IQR) LESP pre (mmHg) 31.4 (15.98, 40.1) vs. post 18.1 (15.4, 31.25); *p* = 0.075, Type II pre 35.64 (22.85, 52.45) vs. post 16.8 (15.98, 26.7); *p* = 0.02, Type III pre 36.05 (16) vs. post 26.7 (6.4); *p* = 0.428], even though not reaching statistical significance for Achalasia subtype I in the RHM group. Data for the 4 s-IRP for the subdivided groups are presented in Table 3.

**Table 3:** Comparison of pre- and postoperative 4 s-Integrated relaxation pressure for the RHM and LHM groups for Achalasia subtypes

RHM	All (*n* = 47)	Type I (*n* = 16)	Type II (*n* = 30)	Type III (*n* = 1)
4 s integrated relaxation pressure (mmHg) preoperative	28.85 (23.635, 36.3)	19.88 (12.68, 32.58)	28.9 (24.7, 39.55)	29.6
4 s integrated relaxation pressure (mmHg) postoperative	14.55 (7.58, 24.6)	17.7 (8.65, 28.35)	14.3 (5.5, 23.05)	19
*p* value	< 0.001	0.317	< 0.001	*

LHM	All (*n* = 31)	Type I (*n* = 16)	Type II (*n* = 13)	Type III (*n* = 2)
4 s integrated relaxation pressure (mmHg) preoperative	25.6 (13.7, 36.3)	22.2 (8, 33.3)	26.7 (20.67, 41.7)	32.35 (16.5)
4 s integrated relaxation pressure (mmHg) postoperative	15.9 (12.45, 23)	16.6 (9.05, 23)	15.2 (13.68, 23.1)	15.1 (4.6)
*p* value	0.019	0.029	0.041	0.315

All values are given in median (IQR) unless stated otherwise

*p* value <0.05 was considered statistically significant

*There was only one patient with Type III achalasia—significance could not be tested

**Fig. 2:**
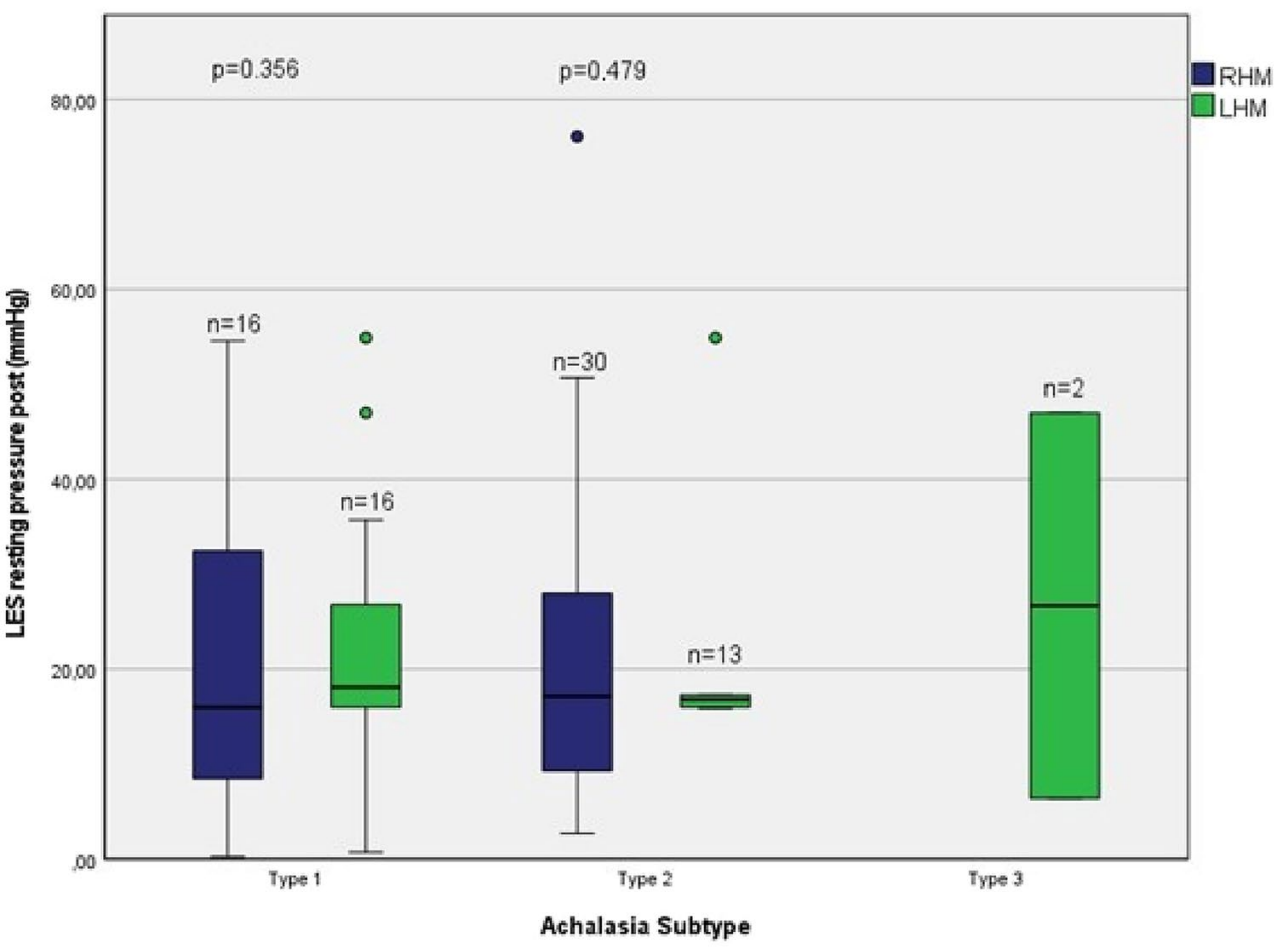
LES resting pressure (mmHg): RHM vs. LHM

**Fig. 3:**
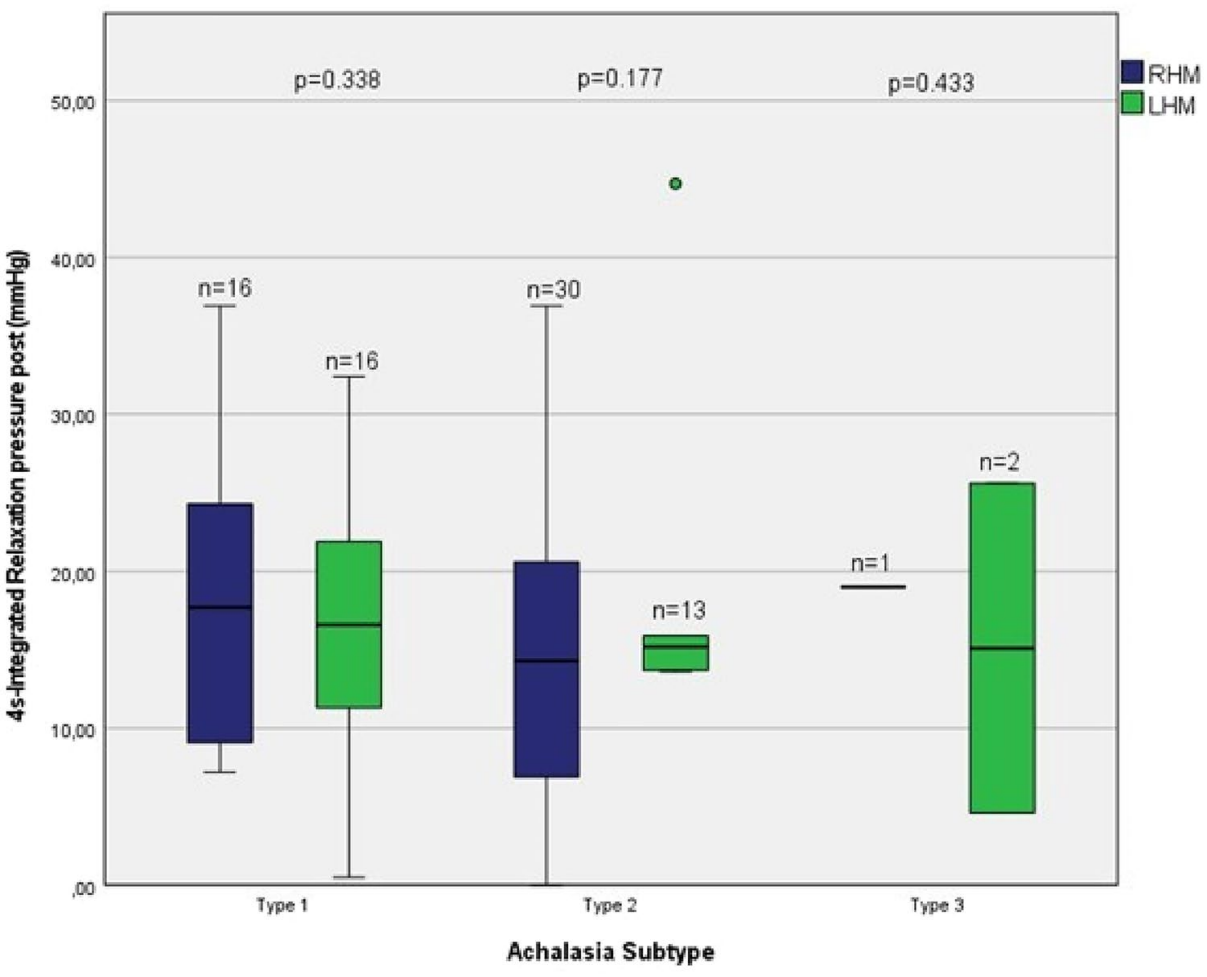
4 s-integrated relaxation pressure (mmHg): RHM vs. LHM

There was no significant difference for the postoperative LESP (*p* = 0.494) and postoperative 4 s-IRP (*p* = 0.323) between these two groups. However, there was no significant difference analyzing the Achalasia subtypes between the RHM and LHM groups either for the postoperative LESP or the postoperative 4 s-IRP (Figs. 2 and 3). As there was only one patient classified with Achalasia type III in the RHM group, respectively 2 patients with Achalasia type III in the LHM group with missing data set for the LESP, the parameters shown here for the sake of completeness with reduced statistical relevance.

## Discussion

With certainty, minimally invasive Heller myotomy with partial fundoplication is the current most effective therapeutic option in patients with symptomatic achalasia shown to be superior to any non-surgical treatment [6, 14]. In recent years with a growing focus on the robotic-assisted approach, studies could demonstrate its safety and efficiency resulting in similar outcomes compared to the laparoscopic approach with an even lower rate of intraoperative esophageal perforations [9–12, 15]. Full thickness perforation of the esophageal junction zone ranges from 8 to 16% for the laparoscopic approach, while it is rarely reported in the robotic-assisted operations [16–18]. While observing no perforations in our robotic-assisted group and only 2 (6.45%) in the laparoscopic cohort, we can substantiate these findings. However, monitoring the success of the surgical treatment, the definition of outcome in these studies is heterogeneous with the majority focussing on clinical symptom scores e.g. the Eckardt Score, GERD-HRQL or a modified Likert-questionnaire [10, 17].

Besides the timed barium esophagogram, the role of high-resolution manometry in the short-term follow-up after surgical intervention is essential to assess the pressure reduction of the lower esophageal sphincter and therefore crucial for evaluation of esophageal emptying [19, 20]. As it is recommended by the American College of Gastroenterologists (ACG) clinical guidelines, functional testing should be preferred over symptom assessment only, as postinterventional symptom improvement may be accompanied by an insufficient pressure reduction of the LES pressure, and therefore, increasing the risk of developing a megaesophagus in the clinical course of these patients [21]. We clearly demonstrated that a significant pressure reduction of the LES resting pressure could be achieved in the robotic-assisted group as well as in the laparoscopic group, indicating a similar functional outcome between the two surgical techniques.

In 2006, Galavani et al. showed a substantially decrease in the LES relaxing pressure in patients who underwent robotic-assisted Heller myotomy [22]. Also Pallabazzer et al. evaluated in 2020 the clinical and functional results in patients who underwent robotic-assisted Heller myotomy with a postoperative follow-up including a high-resolution manometry in a subgroup of 35 of 66 patients, showing a significant reduced postoperative LES relaxing pressure in these patients [11]. However, the follow-up interval was variable and not standardized in these cohorts. Aiming at a half-year period, our median follow-up time was 7 months ranging from 6 to 9 months contributing to a better comparability of the results. Moreover, we could deliver data for the 4 s-integrated relaxation pressure in all patients showing a similar postoperative reduction in both groups. The 4 s-IRP is considered to be the most accurate parameter of deglutitive esophagogastric junction (EGJ) relaxation in patients with achalasia [23], and, thus, serving as one of the diagnostic criteria in manometry according to the current Chicago Classification [4]. As this classification was designed for the diagnosis of primary esophageal motility disorders only, the informative value of these manometric parameters remains interpretative to this point, but several studies revealed that the 4 s-IRP is an excellent parameter to assess EGJ outflow obstruction in the postoperative course in these patients [24, 25].

It should be noted that the surgical approaches were performed sequentially performing the laparoscopic approach from 2015 to 2017 and starting with the robotic-assisted procedure in 2017. Hence, the learning curve by the laparoscopic procedure could be transferred to robotic-assisted operations and therefore may be a factor for less intraoperative perforations and shorter procedure times despite the additional effort with docking and attaching the robotic surgical system.

Our study has several limitations. Regarding its retrospective nature, we were able to obtain a full data set of high-resolution manometric findings and complete clinical symptom score pre- and postoperatively for each individual patient in 62% for all patients investigated only. Since achalasia is a rare disease with non-surgical therapy options, patient enrolment can be challenging contributing to the relatively small number of patients in most studies. Thus, further investigations with larger cohorts are necessary. Since achalasia Type III is the rarest subtype and patients with this subtype are less frequently considered for surgical treatment with the esophageal spasms normally appear above the EGJ, we could only include 3 patients in this subgroup, with is in line with other publications.

In conclusion, robotic-assisted Heller myotomy with anterior Dor fundoplication is a safe and effective procedure with low intraoperative complications compared to the laparoscopic approach. Not only the clinical outcome assessed by the Eckardt Score, but also the functional results measured by distinctive parameters in the high-resolution manometry in short-term follow-up are similar to the laparoscopic procedure and therefore both surgical approaches should be considered equivalent.

## Data Availability

Data are available on demand.
